# Biopsy-based calibration of T2* magnetic resonance for estimation of liver iron concentration and comparison with R2 Ferriscan

**DOI:** 10.1186/1532-429X-16-40

**Published:** 2014-06-10

**Authors:** Maciej W Garbowski, John-Paul Carpenter, Gillian Smith, Michael Roughton, Mohammed H Alam, Taigang He, Dudley J Pennell, John B Porter

**Affiliations:** 1 Haematology Department, University College London Hospitals, London, UK; 2 University College London, London, UK; 3 NIHR Cardiovascular BRU, Royal Brompton Hospital, London, UK; 4 Imperial College London, London, UK; 5 Royal College of Physicians, London, UK; 6 Cardiovascular Sciences Research Centre, St George’s University of London, London, UK

**Keywords:** Thalassemia, Clinical iron overload, Liver iron, Magnetic resonance, Liver biopsy, Calibration

## Abstract

**Background:**

There is a need to standardise non-invasive measurements of liver iron concentrations (LIC) so clear inferences can be drawn about body iron levels that are associated with hepatic and extra-hepatic complications of iron overload. Since the first demonstration of an inverse relationship between biopsy LIC and liver magnetic resonance (MR) using a proof-of-concept T2* sequence, MR technology has advanced dramatically with a shorter minimum echo-time, closer inter-echo spacing and constant repetition time. These important advances allow more accurate calculation of liver T2* especially in patients with high LIC.

**Methods:**

Here, we used an optimised liver T2* sequence calibrated against 50 liver biopsy samples on 25 patients with transfusional haemosiderosis using ordinary least squares linear regression, and assessed the method reproducibility in 96 scans over an LIC range up to 42 mg/g dry weight (dw) using Bland-Altman plots. Using mixed model linear regression we compared the new T2*-LIC with R2-LIC (Ferriscan) on 92 scans in 54 patients with transfusional haemosiderosis and examined method agreement using Bland-Altman approach.

**Results:**

Strong linear correlation between ln(T2*) and ln(LIC) led to the calibration equation LIC = 31.94(T2*)^-1.014^. This yielded LIC values approximately 2.2 times higher than the proof-of-concept T2* method. Comparing this new T2*-LIC with the R2-LIC (Ferriscan) technique in 92 scans, we observed a close relationship between the two methods for values up to 10 mg/g dw, however the method agreement was poor.

**Conclusions:**

New calibration of T2* against liver biopsy estimates LIC in a reproducible way, correcting the proof-of-concept calibration by 2.2 times. Due to poor agreement, both methods should be used separately to diagnose or rule out liver iron overload in patients with increased ferritin.

## Background

The measurement of liver iron concentration (LIC) is clinically useful because LIC reflects total body iron in a predictable way [1]. Changes in LIC over time also reflect total iron balance and hence the efficiency and effectiveness of chelation therapy in controlling total body iron levels [2,3]. Non-invasive estimation of LIC is increasingly used to follow responses to chelation therapy [4]. This is because of the limitations of serum ferritin as a single measure of iron overload and response to chelation treatment, and also because variability in LIC accounts for only 57% of variability in serum ferritin [5]. Serum ferritin may be disproportionately increased relative to LIC by hepatitis and liver damage, generalised inflammation, and by vaso-occlusive syndromes in sickle cell disorders [6]. Conversely, serum ferritin is disproportionately decreased relative to LIC in ascorbate deficiency [7] and in conditions where iron preferentially loads hepatocytes rather than macrophages, such as thalassemia intermedia [8]. Furthermore, the relationship of serum ferritin to LIC, and their trajectories [9], may differ in different chelation regimes [10]. Thus, in the modern management of iron overload, LIC measurement is an important tool. Its clinical applicability increases when it can be performed non-invasively with standardised methodology that is comparable across treatment centres worldwide.

A variety of non-invasive methods have been used to measure LIC including liver susceptometry (SQUID), [11,12], T2-weighted spin-echo MR with [13] or without signal intensity ratios (SIR) to adjacent tissues, [14] and T2* gradient-echo MR with [15] or without SIR, [16-18]. It is important to understand how values derived with these various approaches relate to each other, so that responses to chelation therapy in different studies can be compared and thresholds for treatment intensification can be meaningfully identified.

The T2* MR sequence was developed to measure myocardial iron, but in the first description and initial validation of the use of myocardial T2*, the relation between liver T2* and LIC (measured by biopsy) was documented [16]. The inclusion of the liver analysis at that time was intended to demonstrate a relation between tissue iron concentration and T2*, as myocardial biopsy was impractical and deemed unreliable. At that stage, T2* was not intended or suitable to be used as a standardised method for LIC measurement. Indeed, the curve relating T2* to LIC was known not to reflect a true calibration, because a number of technical issues in the T2* acquisition made it unsuitable for liver iron measurement, the most important factor being that the first echo time (TE) was too long for LIC measurement. An additional factor limiting this first proof-of-concept T2* sequence was that the repetition time (TR) was not constant, which introduced a known error due to the T1 shortening effect of iron. More recently, other MR methods have been introduced specifically for LIC determination, [14,15] of which the R2 Ferriscan technique [14] is currently the most widely used. However, if the T2* method were robustly calibrated for LIC determination, it would be convenient to measure liver and heart iron at the same time using the rapid T2* technique.

Since the initial T2* method was described [16], there have been major improvements in scanner technology and hardware. In the original paper [16], the shortest available TE was 2.2 ms, and 7 further images were acquired with TE ranging up to 20.1 ms, each of which required a separate breath-hold. Using a modern T2* sequence, the shortest TE is typically <1 ms, the TR is held constant for all TE’s, and all images can be acquired in a single breath-hold. This makes the calculation of liver T2* considerably more accurate, especially at higher liver iron concentrations where T2* is very short, eliminates the T1 error, and eliminates mis-registration error between breath-holds. We now report the calibration of liver T2* MR using a state-of-the-art sequence against LIC obtained from paraffin embedded liver biopsies [4,19]. We also report the relation between T2*-LIC values and LIC values obtained by R2 Ferriscan (R2-LIC).

## Methods

### Patients

For the calibration analysis we retrospectively studied 25 patients with transfusional hemosiderosis, including 20 Thalassemia Major, 2 Diamond-Blackfan Anemia, 2 Congenital Sideroblastic Anemia, 1 Pyruvate Kinase Deficiency Anemia, in whom 50 liver biopsies were undertaken at UCLH as part of clinical iron chelation studies on deferasirox; [2,4] patients were also being monitored with annual liver and heart MR scans according to standard clinical management (calibration cohort).

For comparison of T2*-LIC with R2-LIC, 92 scans were performed in 54 patients enrolled in the deferasirox EPIC study [20] and monitored according to standard clinical management with annual liver and heart MR scans (comparison cohort). All patients had transfusional hemosiderosis treated with deferasirox (36 Thalassemia Major, 7 Sickle Cell Anemia, 4 Myelodysplastic Syndrome, 3 Diamond-Blackfan Anemia, 2 Red Cell Aplasia, 2 Pyruvate Kinase Deficiency Anemia).

31 healthy volunteers were invited to participate in the study. IRB approval was granted to the study and all patients and healthy volunteers signed informed consent forms before undergoing scans or liver biopsy. The study was approved by the Royal Brompton and Harefield Research Ethics Committee.

### Liver biopsy

Biopsy LIC was measured in a single central laboratory in Rennes, France (Clinique des Maladies du Foie [Clinic for Hepatic Illnesses], Centre Hospitalier Universitaire) on paraffin embedded sections as previously described [21,22]. Briefly, after obtaining each patient’s consent, a 16G thru-cut needle was passed into the right lobe of the liver under local anaesthesia and aseptic conditions; liver tissue was placed immediately in formaldehyde solution, routinely processed and embedded into wax by local pathology laboratory, batched and sent to Rennes for analysis.

### MR studies

**Figure 1:**
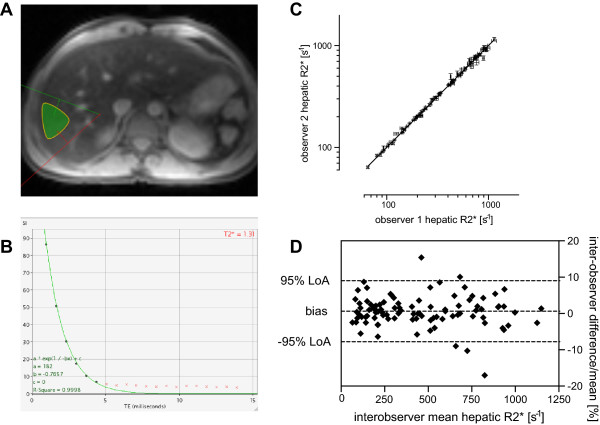
T2* method and inter-observer reproducibility. (A) A transverse slice of the liver where an example of the region of interest (ROI) is seen in green within the mid/lateral area free of large vessels. **(B)** T2* estimation using the truncation method in exponential curve fitting: crosses represent truncated data points whereby only the first 5 points contribute to the decay curve model. **(C)** The inter-observer reproducibility and agreement for R2* between observer 1 and 2 (both in triplicate, error bars show SEM, 96 scans) with line of identity. Coefficient of variation 5.79%. **(D)** Bland-Altman analysis of inter-observer liver R2* percentage differences plotted against the mean R2* of the two observers. The *bias* as the mean of all differences was 0.61 ± 4.29% (SD). The *95% limits of agreement (*LoA) were between -7.79 and 9.02%.

All MR scans were performed on a 1.5 T Sonata MR scanner (Siemens, Germany) using a 4-channel anterior phased array coil at Royal Brompton Hospital, London (RBH). A transverse slice through the centre of the liver was imaged using a multi-echo single breath-hold gradient echo T2* sequence with a range of echo times (TE 0.93-16.0 ms). T2* was measured using Thalassemia Tools (Cardiovascular Imaging Solutions, London, UK) from a region of interest (ROI) in an area of homogeneous liver tissue, avoiding blood vessels and other sources of artefact. To correct for background noise, a truncation method was used for curve fitting [23] (Figure 1A-B). All T2* measurements were performed in triplicate by two independent observers choosing three separate ROIs to analyse. The ROIs were chosen to be as large as possible in three separate areas of the liver (anterior, mid/lateral and posterior). The observers were blinded to each other’s and to liver biopsy results. T2* is conventionally expressed in milliseconds [ms], while the units of its reciprocal R2* are s^-1^ with R2*[s^-1^] =1000/(T2*[ms]). Liver R2* in healthy volunteers (n = 31) was 37.0 ± 1.1 s^-1^ (mean ± SEM), SD 6.1 s^-1^, range 28.7 to 54.4 s^-1^.

R2-LIC (Ferriscan) sequences were obtained on the same scanner according to the EPIC study protocol, as reviewed recently [24].

### Statistical analysis

Replicated measurements were averaged for regression purposes however treated separately to estimate inter-observer reproducibility. For inter-observer liver R2* agreement, 96 liver R2* scans from 38 patients were independently evaluated in triplicate by two observers as described above (Figure 1) and analysed using Bland-Altman plots [25]. The degree of relationship between log-transformed biopsy LIC and liver R2* or T2* was estimated by Pearson correlation method. Mixed model linear regression was used with patients declared as random effects and predictor variables as fixed effects. Where the effect of nesting of the data within patients was insignificant (by a likelihood ratio test), ordinary least squares regression (LSR) was used to fit the model line with 95% prediction bands. P-value <0.05 was assumed statistically significant. Statistical analysis was performed using GraphPad Prism version 6.0 for Mac, GraphPad Software, San Diego California USA, http://www.graphpad.com, and STATA Ver 10.1 (Stata Corp, College Station, TX).

## Results

### Reproducibility and predictability of R2*

On difference-versus-mean Bland-Altman analysis (Figure 1), there was good inter-observer agreement, with increasing deviation from zero for R2* > 500 s^-1^. Bias was negligible at 0.61%, 95% limits of agreement (LoA) were approximately 8 and 9% below and above the bias, corresponding to about 1.5 mg Fe/g dw in each direction (see Equation 1). This was considered clinically acceptable, especially for mean liver R2* values <400 s^-1^ where the inter-observer agreement was robust with the spread nearly limited to the 95% CI of the bias only.

### Relationship of T2* to LIC

**Figure 2:**
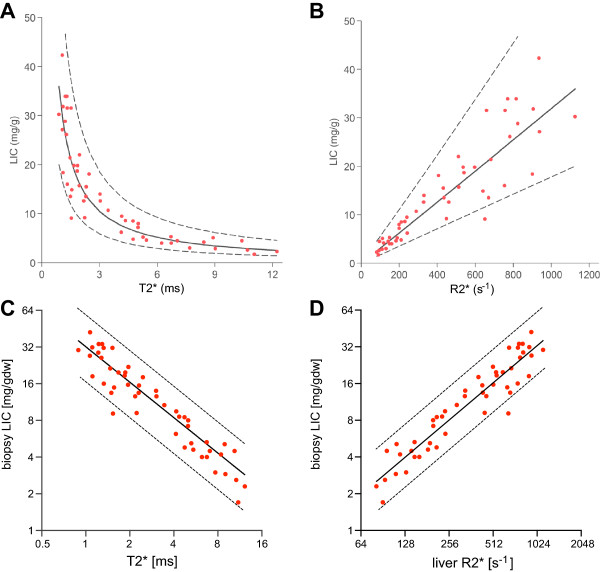
The calibration of T2* and R2* models for LIC estimation. (A) The relationship of liver T2* to LIC obtained by biopsy in 50 samples. The regression fit from Figure 2C was plotted onto the data after exponentiation: LIC = 31.94(T2*)^-1.014^ with 95% CI 27.8 to 36.7 (87-115%) for the first term and -1.118 to -0.91 (110-90%) for the exponent. **(B)** The relationship of liver R2* (=1000/T2*) to LIC of the data in 2A, with regression line from the model in 2D after exponentiation: LIC = 0.029R2*^1.014^; 95% CI 0.016 to 0.054 (55-186%) for the first constant, 0.910 to 1.118 (90-110%) for the exponent. **(C)** Log-log plot of LIC versus liver T2*: Pearson r = -0.94 (95% CI -0.97 to 0.91, p < 0.0001); linear regression: ln(LIC) = 3.464-1.014ln(T2*); slope SE = 0.052, 95% CI -1.118 to -0.9 (110-90%); intercept SE = 0.069, 95% CI 3.325 to 3.603 (96-104%), r squared 0.89. **(D)** Log-log plot of LIC versus liver R2*: Pearson r = 0.94 (95% CI 0.90 to 0.97, p < 0.0001); linear regression: ln(LIC) = 1.014ln(R2*)-3.54; slope SE = 0.052, 95% CI 0.910 to 1.118 (90-110%); intercept SE = 0.30, 95% CI -4.152 to -2.928 (117-83%); r squared 0.89.

In total, 50 data pairs (biopsy LIC value and corresponding liver T2*) were available for analysis on 25 patients. Median biopsy-to-scan window was 78 days (IQR 39–160, range 2–228). LIC values ranged from 1.7 to 42.3 mg/g dw (median 12.6). Nesting effect of patients was insignificant and did not warrant a mixed model regression approach. T2* and LIC showed curvilinear relationship (Figure 2A), however assumptions of distribution and variance of residuals were not met on LSR. Therefore further analysis was performed after log-transformation of both variables (Figure 2C) which were highly correlated with Pearson r = -0.94. Linear LSR lnLIC = 3.464-1.014ln(T2*) was exponentiated to a non-linear model LIC = 31.94(T2 *)^- 1.014^ (Equation 1) with r-squared = 0.89, which for 5 ms corresponds to 6.24 mg/g dw (95% CI 5.67 to 6.88 or 91-110%) and for 2 ms to 15.82 mg/g dw (14.46 to 17.31 or 91-109%). Therefore the early T2* method [16] estimating LIC at 6.86 mg/g dw for T2* of 2 ms should be corrected by an average factor of × 2.2 + 0.57 to obtain values comparable with present calibration.

### Relationship of R2* to LIC

With T2* values expressed as R2* (=1000/T2*), the relationship initially looked linear, however non-normally distributed residuals and heteroscedasticity (fanning-out effect) prevented both linear and non-linear LSR analysis (Figure 2B). Log-log transformed data allowed for a linear LSR model (Figure 2D), which for an R2* of 200 s^-1^ gave the same best fit and 95% confidence interval (CI) LIC value as its T2* equivalent of 5 ms in the T2* model. Because parameter estimation by unconstrained regression showed larger standard error of the intercept than in the T2* model, further analysis was performed using the calibration Equation 1. Due to the nature of the reciprocal relation between T2* and R2*, a greater intercept error is introduced when R2* is derived from T2* and compared to liver iron. For this reason, we have chosen to use T2* for our calibration.

LIC did not relate to biopsy grading (p = 0.15, one-way ANOVA), but showed a weak significant relationship between LIC and staging of fibrosis (p < 0.01, one-way ANOVA with post-test for linear trend relating stage (1–5) to mean LIC: slope 1.99, r-squared 0.18, p < 0.001). In view of the latter, we examined whether exclusion of stage 4 and 5 data pairs altered the calibration equation, however differences in absolute sum of squares for both models were insignificant (p > 0.82) therefore no changes to Equation 1 were made (data not shown).

We have also examined whether excluding data pairs with large biopsy-MR window alters the LIC to T2* relationship, and have therefore compared a linear model based data with a median window of 28 days (IQR 14–45, range 2–56, n = 17) with Equation 1, however there was no significant difference between absolute sum of squares (p = 0.06, data not shown).

### Relationship of new T2* (R2*) calibration to R2 (Ferriscan) LIC measurements

The LIC values obtained with the above recalibrated T2* method were compared with LIC values obtained using the R2 (Ferriscan) method in 92 scans performed in 54 patients. The median time window between the T2* and R2 scan was 13 days (range 0 to 91). R2-LIC values were measured across the entire clinically relevant range from 1.0 to 43.3 mg/g dw (median 11.95, IQR 5.50 to 21.45). T2*-LIC values ranged from 1.25 to 41.10 mg/g dw (median 12.20, IQR 4.65 to 19.75). Data pairs were also restricted to those obtained within 30 days and the two data sets compared (not shown), however there was no significant difference in the regression line or the data scatter between the two sets.

**Figure 3:**
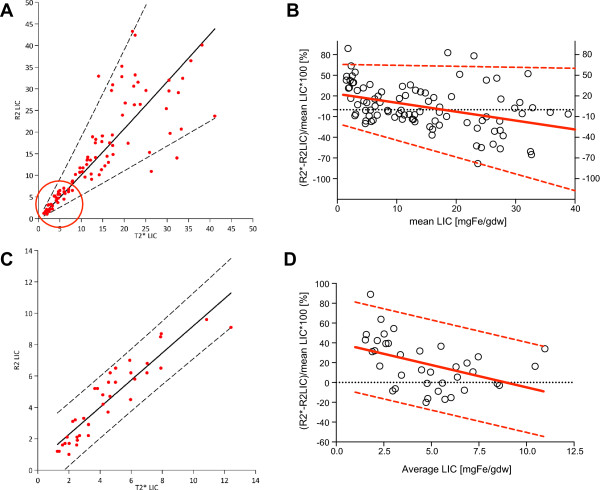
Relationship of new T2*-LIC to R2-LIC (Ferriscan) measurements. (A) R2-LIC plotted against T2*-LIC (derived from Equation 1) for comparison cohort; range with lowest scatter circled. Mixed model regression on whole range log-transformed data ln(R2-LIC) = 1.04×ln(T2*-LIC)-0.08, with slope and intercept 95% CI of 0.96 to 1.11 (p < 0.001) and -0.26 to 0.11 (p = ns), is shown here exponentiated to R2-LIC = 0.83×T2*-LIC^1.04^ with 95% CI 0.96 to 1.11 and 0.55 to 1.29, r squared = 0.65. **(B)** Whole range Bland-Altman plot of percentage difference vs mean LIC with non-uniform scatter with linear regression correction. Corrected bias is -1.29*(mean LIC)+23.03; absolute residuals R were related to LIC: R = 0.47*(mean LIC)+17.44, p = 0.01, therefore, accounting for growth in variance, 95% LoA were from -0.14*(mean LIC)+65.9 to -2.44*(mean LIC)-19.8 or changing from ±45 to ±90% across the whole range. **(C)** Mixed model linear regression of R2-LIC on T2*-LIC for R2-LIC range 0–10 mg/g dw: R2-LIC = 0.87 × R2*LIC-0.55, slope 95% CI 0.74 to 0.99 (p < 0.001), intercept -0.01 to 1.19 (p = 0.089); r squared = 0.86. Insignificant intercept can be abandoned to give a proportionality with a slope of 0.96, 95% CI 0.89-1.02, p < 0.001, which being indistinguishable from 1, follows the line of identity. **(D)** Bland-Altman plot of the mean of the two methods (x-axis) plotted against their percentage difference (y-axis) for the range marked by circle in 3A, which for R2-LIC values <10 mg/g dw (here in 37 scans on 26 patients) shows non-uniform scatter corrected by linear regression. Corrected bias is -4.51*(mean LIC)+40.23, p = 0.007, SD of residuals 23.22%. Absolute residuals did not relate to LIC (p = 0.99), therefore 95%LoA were bias ± 1.96*23.22% or ±45.51%.

Figure 3 shows the relation between R2-LIC (Ferriscan) and T2*-LIC derived from Equation 1. With heteroscedastic data scatter (Figure 3A), the *whole range* relationship could not be described by linear LSR unless the data were log-transformed. Mixed model regression of the log-transformed data (significant patient nesting effect p < 0.001) showed ln(R2-LIC) = 1.04 × ln(T2*-LIC)-0.08, exponentiated to (R2-LIC) = 0.93 × (T2*-LIC)^1.04^. The best-fit line closely follows the line of identity (95% CI of both constants includes 1), however confident prediction of higher values is impossible due to increasing scatter, which is supported by low explained variance (r-squared = 0.65) and lack of agreement in the *whole range* Bland-Altman plot (Figure 3B). Low range data however, shows that the scatter is relatively limited (Figure 3A red). Consequently, better agreement between the two methods is obtained at LIC < 10 mg/g dw (here further analysed in 37 scans on 26 patients), with a small bias of 0.65 and 95% LoA from -1.31 to 2.56 mg/g dw (not shown). This however corresponds to ±45% on percentage difference Bland-Altman plots after linear correction for non-uniform scatter (Figure 3D). For the data-points defined by R2-LIC <10 mg/g dw (see Figure 3A red circle), there does appear to be a much clearer, linear relationship using the values on their original (untransformed) scales. The regression diagnostic plots (not shown) and comparison of the original and logged data graphs (Figure 3A, C), suggest the prediction intervals for the original data to be a better fit. Mixed model linear regression on the limited range data (nesting effect p = 0.012) gives (R2-LIC) = 0.87 × (T2*-LIC) + 0.55 (equation 2) with r-squared = 0.86. Insignificant intercept (p = 0.09, 95% CI -0.09 to 1.19) allows forcing the model through zero, which leads to a proportionality slope of 0.96 (95% CI 0.89 to 1.02, p < 0.001) for liver T2*-LIC with no significant change to r-squared (0.84), or of 0.03 for liver R2* only (<400 s^-1^). Given that the slope is indistinguishable from 1, this essentially shows an identity relationship between the two methods within the limited range. However percentage difference Bland-Altman plot for this range still shows poor agreement even after linear correction for non-uniform scatter (±45%, Figure 3D).

The inter-observer repeatability coefficient (IRC) for T2*-LIC was 0.37 mg/g dw over this range (0-10 mg/g dw), as calculated from within-subject variance (one-way ANOVA with patient as group) [25]. IRC can be compared with the interval between 95% LoA on difference Bland-Altman plot of the two methods (not shown) such that ±0.37 i.e. 0.74 versus 3.87 (from -1.31 to 2.56 mg/g dw) would account for approximately 19% of the variability represented by the range between 95% LoA.

## Discussion

Non-invasive assessment of LIC is increasingly used as an alternative to biopsy and in order for clinicians to have evidence-based guidelines for the management of iron overload, it is important that LIC measurements are comparable across studies, treatment centres and continents. Recently, considerable effort has been undertaken to standardise the assessment of myocardial iron with T2* across centres internationally [23,26], but knowledge about how different measures of LIC compare is relatively limited. It is convenient for patients and clinicians if LIC can be assessed by MRI at the same time as myocardial T2* estimation, and this is possible in principle using T2* methodology. However the measurement of LIC by T2*, as originally described in 2001, [16] was undertaken as a proof of concept: namely that tissue T2* was related to tissue iron concentrations. This was an early MR technique, and not in any way optimised for the liver, where high iron concentrations cause low T2* values requiring very short minimum echo-times for accurate measurement. Such short echo times were not achievable with typical MR technology in 2001. However, since that time, it has become clear that a calibration of liver T2* is required which uses current improved MRI methodology and which allows comparisons with other available techniques.

A less widely appreciated reason for why further calibration is necessary is that biopsy LIC, the so-called ‘gold standard’ for LIC determination, is also not standardised and highly variable. The variability of needle biopsy LIC is dependent on the biopsy specimen size [27] (CV of approximately 19% when <4 mg/g dw [28] and 9% when >9 mg/g dw [29]), on fibrosis content, [27] and the presence of cirrhosis (CV >40%) [28,30]. Biopsy LIC determination also varies considerably between laboratories due to differences in the real or perceived ratios of wet-to-dry weights of tissue samples; e.g. the mean wet-to-dry weight ratio for vacuum-dried fresh tissue was 3.76, but up to 6.26 in paraffin-embedded de-waxed tissue, [22] resulting in a corresponding difference in the measured dry tissue iron concentration. In the original T2* publication by Anderson, [16] biochemical determination of LIC was performed on formalin fixed cores but not on paraffin embedded tissue, [29] which could explain in part why there is an approximately 2-fold difference with the calibration in this paper where paraffin embedded biopsies were used. Such differences have also been an issue with SQUID; in a recent large-scale study an approximately two-fold correction factor was required between biopsy and SQUID methodology [2,4].Interestingly, the calibration of T2* in this paper, gives values close to those obtained with the R2 technique, however agreement between both methods remains poor over the whole range of measurements. As scatter was non-uniform, showing lower stable variance and the proportionality slope indistinguishable from 1 in the low range of measurements (Figure 3D), we wished to examine whether method agreement improves if comparison is limited to that range. However limits of agreement in this range are still unacceptable at ±45%.

Because the calibrations in this paper were retrospective, utilising MRI data obtained during routine testing, and biopsy samples obtained as part of chelator studies, [2,4] the timing of biopsies relative to MRI scans was not necessarily coordinated so that the biopsy-to-scan intervals were variable. However, no significant difference was found between a model based on narrow biopsy-to-scan windows and that described by Equation 1. The most likely explanation of this finding is that, as chelation-induced difference in LIC over time increases with biopsy-to-scan window and may therefore cause T2* to overestimate or underestimate LIC (by preceding or following biopsy, respectively), the positive and negative time windows distribute randomly around zero, and their estimation bias is effectively cancelled out (data not shown). Otherwise one would have to postulate long-term stability of LIC, which is likely not to be the case.

We have attempted to examine the reproducibility of the current T2* (R2*) methodology at a single centre (RBH). This has been achieved with two independent observers, each choosing the ROI to examine in triplicate, and by then comparing the intra-observer mean liver R2* between the two observers (inter-observer reproducibility, IOR). This IOR is acceptable over a wide range of R2*, being particularly good for R2* < 500 s^-1^ or LIC ≈ 15 mg/g dw (see Figure 1C-D). Other reproducibility estimates were beyond the scope of this study, as it relied on the analysis of data generated either as a part of routine monitoring of patients, or from scans obtained during the assessment of new chelation therapies [2,4,24]. Such studies would be of value if this liver T2* technique was to be used in those international centres where myocardial T2* has already been validated [23,26].

IRC-derived variability of T2*-LIC constitutes 19% of the variability between R2-LIC and T2*-LIC represented by the 95% LoA interval (Figure 3D). Other sources of variability related to repeatability and reproducibility are unlikely to explain the poor agreement between both methods as reproducibility studies for T2* [26,31,32] and R2 LIC indicate (excellent single centre inter-scan [14], although not multi-centre with broad LoA -71 to 74% [33]). Choice of ROI is also not likely to reduce agreement as inrer-slice reproducibility is high [14,17] and the magnitude of significant increase in LIC variability from small-ROI vs whole-liver methods although significant is practically too small [34] to explain the poor agreement we and others have observed. Non-repeatability-related variability inherent in both methods, e.g. associated with different sensitivity to noise of both methods, to iron particle size, clumping, distribution and resultant proton mobility [35] within tissue is perhaps a more likely reason for lack of within-patient agreement. Wood and colleagues have recently shown that, when viewed separately, both methods adequately reflect absolute levels and trends in LIC in patients with iron overload on chelation therapy [36,37], but the fact that they cannot be used interchangeably due to poor cross-sectional agreement (±50%), despite excellent concordance of chelation efficiency estimates derived from them, all point to a possibility that methods access slightly different pools of iron (e.g. hepatocyte vs macrophage, haemosiderin at different stages of maturation) which nonetheless change in parallel during chelation or that they sample them in a different way. Differences in acquisition methods may also affect agreement (see Table 1). The R2 Ferriscan method uses a spin echo sequence, [14] unlike the gradient echo sequences used for T2* in this paper. This decreases the effect of surrounding tissue on the signal but also has a non-linear relationship to LIC particularly at high LIC values. In the MRI technique described by Gandon, [15] which used a T2-weighted sequence where liver values were related to the signal from skeletal muscle, a linear relationship of biopsy LIC to MRI SIR was shown up to approximately 29 mg/g dw. In the R2* method published by Wood, [17] LIC derived from R2* values are broadly similar to T2*-LIC values obtained in our paper: an R2* of 300 s^-1^ predicted a mean LIC of 8 mg/g dw with 95% prediction bands from approximately 3 to 12 mg/g dw, while for 600 s^-1^ 15 (9–20)mg/g dw, respectively [17]. Hankins [18] examined LIC <30 mg/g dw and from their Figure 1A it can be observed that, similarly to *Wood et al.*, 300 s^-1^ relates to mean LIC of 8 mg/g dw, with prediction bands between 4.5-12, while for 600 s^-1^ – to 16.5 (12.5-20) mg/g dw.

**Table 1:** Comparison of LIC calibration methods

**Authors**	**Method**	**Liver slice thickness**	**ROI size**	**ROI number**	**Localization within liver**	**TE, step [ms]**	**TR [ms]**	**Breath-hold**	**Background noise reduction**	**Notes**
**St Pierre et al.**[14]	T2	5 mm, 5 mm gap	Whole liver	1	Largest axial slice	6-18, 3	2500	Single	Voxel intensity smoothing	SSE
**Wood et al.**[17]	T2*	15 mm	Whole liver	1	Mid-hepatic slice	0.8-4.8, 0.25	25	Single	Variable offset	Single echo gradient echo
**Wood et al.**[17]	T2	15 mm, 5 mm gap	Whole liver	4	Entire liver boundary without obvious hilar vessels	3.5-30, nd	nd	Single	Variable offset	Single echo 120°-120° Hahn echo
**Hankins et al.**[18]	T2*	10 mm	Small, variable	1	Transverse slice, at the level of main portal vein origin, excluding vessels and bile ducts	1.1-17.3, 0.8	nd	One per TE	Truncation	Multiecho gradient echo
**McCarville et al.**[34]	T2*	10 mm	Whole liver	1	Transverse, at the level of main portal vein origin	1.1-17.3, 0.8	200	Single	nd	Multiecho gradient echo
**McCarville et al.**[34]	T2*	10 mm	Small, variable	1	Right lobe, excluding vessels and bile ducts	1.1-17.3, 0.8	200	Single	nd	Multiecho gradient echo
**Gandon et al.**[15]	T2*	10 mm	Small, variable	3	Right lobe	4-21, nd	120	nd	Saturation threshold defined	GRE
**Garbowski et al.**	T2*	10 mm	Small, variable	3	Transverse mid-hepatic slice right lobe, excluding vessels and bile ducts	0.93-16, nd	nd	Single	Truncation	Multiecho gradient echo

All scanners are 1.5 T; TE, echo time; TR, repetition time; SSE, single echo spin echo; GRE, gradient recalled echo; ROI, region of interest; nd, no data.

**Figure 4:**
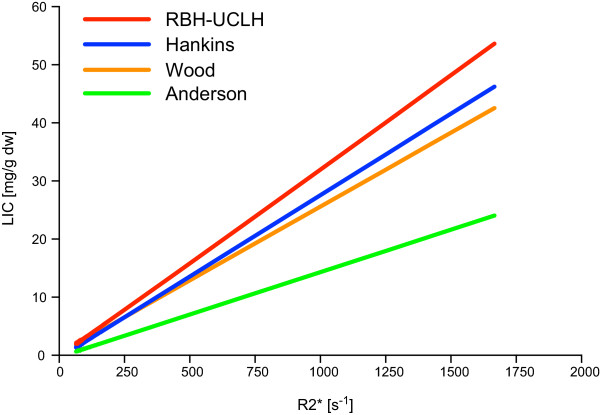
Slope comparison of R2* LIC calibration models. Slope comparison of R2* based LIC calibration methods (after J. Hankins et al. [18]): by L. Anderson LIC = 0.0146(R2*)-0.27 (green), by J. Wood LIC = 0.0254(R2*) + 0.202 (orange), by J. Hankins LIC = 0.028(R2*)-0.45 (blue), our method (RBH-UCLH) LIC = 0.032(R2*)-0.14 (red).

Our data show higher LIC values for similar R2* than those quoted in the above two papers (Figure 4) and the reason for this is at least twofold. Treatment of biopsy specimens differed, with our samples washed in xylene before embedding in paraffin, which dissolves lipids, reduces dry weight and increases iron-to-weight ratio more than when fresh samples are dried before iron quantification [38] as in Wood and Hankins studies. Furthermore, the offset model used by Wood for R2* measurement [17] is different from our truncation model used in this study, whereas truncation model used by Hankins is really an offset model with direct noise floor subtraction based on Anderson’s early model. We have demonstrated [39] that, compared with the truncation model, the offset model tends to overestimate R2* due to its technique for noise compensation. For this particular reason, if R2* is measured using our truncation model, lower LIC values are expected than if calculated by using the calibration equations from Wood or Hankins. This finding also highlights the importance of using a calibration equation appropriate to the analysis technique to get an accurate LIC estimation in clinical practice.

## Conclusions

In conclusion, the R2* method described in this paper shows near-linearity to biopsy LIC values over a wide clinical range. There is good inter-observer reproducibility, however it is not possible to know to what extent the decrease in reproducibility at high LIC values is a consequence of the inhomogeneity in liver iron obtained by biopsy and to what extent this is a reflection of the T2* measurements, but this phenomenon has also been noted with the R2 Ferriscan technique and SQUID. Differences from other R2*/T2* derived LIC calibrations may be explained by differences in post-biopsy sample processing and fitting algorithms. Poor agreement between T2*LIC and R2LIC, even at low magnitude is not derived from ROI- and reproducibility-related variability of both methods but likely stems from different sensitivity of R2 and R2* to iron distribution and noise sources.

## Abbreviations

CI: Confidence interval; CV: Coefficient of variability; dw: Dry weight; IOR: Inter-observer reproducibility; IQR: Inter-quartile range; IRB: Institutional Review Board; IRC: Inter-observer repeatability coefficient; LIC: Liver iron concentrations; LoA: Limits of agreement; LSR: Least squares regression; MR: Magnetic resonance; RBH: Royal Brompton Hospital; ROI: Region of interest; SD: Standard deviation; SEM: Standard error of the mean; SIR: Signal intensity ratio; SQUID: Superconducting quantum interference device; TE: Echo time; TR: Repetition time; UCLH: University College London Hospitals.

## Competing interests

MG, JC, MR, MA, JP declare no relevant conflicts of interest. GS has consultancy agreement with Novartis. TH has received honoraria from Novartis, Apotex and AMAG, DP is a director and stockholder of Cardiovascular Imaging Solutions, London, UK and a consultant to Novartis, ApoPharma, Siemens, Bayer, AMAG and Shire.

## Authors’ contributions

MG, DP and JP designed the study, and wrote the manuscript. MG recruited subjects and analysed the data. JC, GS, MA, and TH performed and analysed the scans, and reviewed the manuscript. TH developed the T2* measurement model. MR (study statistician) performed the regression analyses and reviewed the manuscript. All authors read and approved the final manuscript.
